# Cervical and systemic concentrations of long acting hormonal contraceptive (LARC) progestins depend on delivery method: Implications for the study of HIV transmission

**DOI:** 10.1371/journal.pone.0214152

**Published:** 2019-05-16

**Authors:** Lyndsey R. Buckner, Erma Z. Drobnis, Molly S. Augustine, Lynette K. Rogers, Jill Akers, Patricia D. Mott, Thomas J. Hope, Alison J. Quayle, Danny J. Schust

**Affiliations:** 1 Department of Microbiology, Immunology and Parasitology, Louisiana State University Health Sciences Center, New Orleans, LA, United States of America; 2 Department of Obstetrics, Gynecology, and Women's Health, University of Missouri, Columbia, MO, United States of America; 3 The Research Institute at Nationwide Children’s Hospital, The Ohio State University, Columbus, OH, United States of America; 4 Cell and Molecular Biology, Northwestern University, Chicago, IL, United States of America; Boston University, UNITED STATES

## Abstract

Progestin-only long-acting reversible contraceptives (LARCs) are increasingly popular among women seeking contraception; however, recent epidemiological studies suggest that systemically administered medroxyprogesterone acetate (MPA) may increase HIV acquisition. In order to determine the exact mechanisms underlying increases in transmission specific to MPA use and to test safer, alternative contraceptive progestin types and delivery methods, *in vitro* modeling studies must be performed. To achieve this, it is imperative that accurate hormone concentrations be utilized when modeling progestin-mediated outcomes, as the down-stream effects are dose-dependent. The local concentrations of progestins to which the lower female genital tract tissues are exposed after initiation of LARCs are unknown, but they likely differ from peripheral concentrations, dependent upon the progestin type and delivery method. Here, we measured *in vivo* endocervical and plasma concentrations of (1) systemically-delivered depo MPA (DMPA), (2) levonorgestrel (LNG) delivered via intrauterine system (IUS) and (3) etonogestrel (ETG) delivered via vaginal ring in women who recently initiated contraception treatment. Levels of ETG and LNG in cervical secretions were 100–200 fold higher than plasma levels. In contrast, measurable MPA levels were approximately 10-fold higher in plasma compared to cervical secretions. These results will inform the design of accurate *in vitro* studies on the influence of progestins on epithelial cells, tissue explants, and peripheral blood cells, to be able to better predict *in vivo* outcomes. Subsequent observations will aid in determining how MPA might influence HIV acquisition and may facilitate identification of optimal progestin-containing LARC alternatives for women at high risk for HIV infection.

## Introduction

Long-acting reversible contraception (LARC) options, particularly injectables and intrauterine devices, are becoming increasingly popular in developed and under-resourced countries due to their discrete nature, high efficacy, and low user-failure rates [1]. However, over the past decade, a number of studies have reported that certain LARCs, particularly medroxyprogesterone acetate (MPA) delivered as the systemic injectable depo MPA (DMPA), can increase women’s susceptibility to human immunodeficiency virus (HIV) infection [2–8]. In response, the World Health Organization (WHO) has recently recommended that alternatives to DMPA should be considered for women at high risk for HIV infection who desire contraception [9]. The mechanisms underlying increased HIV acquisition in DMPA users are not entirely known, but it is important to better understand such mechanisms to identify alternative LARCs that may be safer options for high risk women.

To determine the mechanism by which MPA influences HIV transmission and to identify better contraceptive options for women at high risk for HIV acquisition, *in vitro* studies are required using cells and/or tissues from the female genital tract (FGT) where heterosexual transmission of HIV occurs. There are several factors that complicate accurate modeling of hormonal influences on the FGT during HIV transmission and the translation of *in vitro* observations to *in vivo* outcomes. For instance, several types of progestins with disparate receptor binding capacities [10–12] are included in modern LARCs. It has been hypothesized that adverse HIV transmission outcomes associated with DMPA use might be secondary to non-contraceptive, immunosuppressive effects mediated by high binding affinity to the glucocorticoid receptor (GR) [13–17]. Other LARCs currently on the market, such as levonorgestrel (LNG), delivered via an intrauterine system (IUS), and an etonogestrel (ETG)/estradiol combination, delivered via vaginal ring, exhibit low cross-reactive GR binding affinities when compared to MPA and such differences may [18–22] explain why increased HIV acquisition has not been associated with their use in females to date.

Many of the effects of steroid hormones on epithelial immune and barrier functions are concentration-dependent [23, 24], and there are several routes of delivery for progestin-containing LARCs. Both likely affect infection risk *in vivo*, and both complicate modeling infection susceptibility *in vitro*. Most *in vitro* studies are performed using serum levels of contraceptive progestins, which are well documented [25–28]. It is likely, however, that serum levels of progestins such as LNG and ETG that are delivered locally via IUS and vaginal rings, respectively, do not accurately reflect the local levels to which the FGT epithelial cells and underlying tissues are exposed. Indeed, it has been reported that endometrial levels of LNG delivered via IUS are significantly higher than circulating levels of the progestin [29]. It is likely that differences in local *vs*. systemic progestin levels will alter immune responses *in vivo*, as the production of immune mediators, such as cytokines and anti-microbial peptides, are known to be progestin dose-dependent [28, 30].

The objective of this study, therefore, was to determine the cervical concentrations of systemically-delivered MPA, locally delivered LNG (via the LNG-IUS) and locally delivered ETG (via vaginal ring) and to compare these local concentrations to their peripheral blood counterparts. We chose to investigate progestin levels in the cervix because it is an important site for HIV transmission, and the cervical epithelium generates many innate immune mediators [31, 32] that can be modified by endogenous and exogenous hormones, including progestins [31, 32]. It has been reported that natural endogenous progesterone is 10 fold lower in cervical mucus compared to serum levels; however, data does not exist on the levels of exogenous progestins within fluids from the lower FGT following systemically and locally administered contraceptives [33]. Because progestin-mediated effects are concentration dependent, determining progestin levels at these local sites will inform *in vitro* studies that use FGT tissue or epithelial cell modeling and allow more accurate investigation of specific LARC progestin subtypes and delivery methods. Investigations using physiologically relevant hormone exposure parameters to study site- and progestin-specific effects *in vitro* should help us to provide more informed clinical contraceptive choices for women at high risk for HIV infection.

## Materials and methods

### Participant enrollment

Institutional Review Board approval for this study was obtained from The University of Missouri (MU) Institutional Review Board (ID# FWA00002876). Participants were women attending the MU Outpatient General Obstetrics and Gynecology clinic who requested long acting progestin-containing contraception and who presented for treatment with either (1) MPA delivered systemically (Depo-provera, 104 mg), (2) levonorgestrel (LNG) delivered via an intrauterine system (Mirena, 52 mg) or (3) etonogestrel (ETG) delivered via vaginal ring (NuvaRing, 11.7 mg etonogestrel and 2.7 mg ethinyl estradiol). Women who satisfied inclusion and exclusion criteria as described below who were willing to participate in the study were consented and compensated for their time. The enrollment eligibility criteria included healthy females aged 16–35 presenting for initiation of one of the above LARCs. Exclusion criteria were: (1) use of hormonal contraception within 8 weeks prior to enrollment, (2) pregnant or delivered within 5 weeks of enrollment, (3) untreated cervical dysplasia or treatment of cervical dysplasia within 3 months of enrollment, and (4) history of immune-mediated disease or immunotherapy. If a participant did not return for a second visit her samples were excluded from the primary study analyses.

### Sample collection

Upon enrollment, samples were collected from patients at their baseline visit prior to progestin treatment. The same samples were then collected approximately 1 month following treatment initiation as the post-treatment assessment. For some patients on LNG, a third sample was collected between 3 and 6 months post-progestin initiation.

At each sample collection visit, vaginal samples for standard assessment of *Chlamydia trachomatis*, *Neisseria gonorrhoeae*, bacterial vaginosis (BV), yeast, leukocytes and sperm were obtained, as previously described [34]. In addition, cervical secretions were collected using Merocel ophthalmic sponges (Medtronic Xomed, Inc., Jacksonville, FL) [35]. Two ophthalmic sponges were placed in the cervical os for 2 minutes to collect cervical secretions. Sponges were then immediately placed in a -80°C freezer until eluted for batched hormone quantitation assays. Five mL of blood was collected for serum separation and serum aliquots were frozen at -80°C until use. Cervicovaginal mucus (CVM) was collected via an Instead Softcup (The Flex Company) that was placed in the vagina for 30 seconds and then removed. Softcups were then placed inside a 50mL conical tube and centrifuged at 750xg for 10 minutes at 4°C to collect CVM [36].

### Ophthalmic sponge elution

Known quantities (5 ng, 50 ng, 500 ng) of progesterone (Sigma Aldrich, St. Louis, MO) in PBS were initially absorbed onto Merocel (Medtronic Xomed, Inc., Jacksonville, FL) ophthalmic sponges for 10 minutes at room temperature. Progesterone (50 ng, 500 ng) was also added to 25 uL cervicovaginal mucus collected via Instead Cup from three normally cycling subjects prior to initiation of any progestin treatment. Progesterone spiked mucus was absorbed onto sponges in a manner similar to that used for samples containing progesterone alone. Sponges were frozen at -80°C. Prior to elution, all sponges were thawed on ice. To investigate which elution buffer optimized progesterone extraction, 300 uL of water or 30%, 70%, or 100% methanol mixtures in water were added to replicate sponges for 10 minutes on ice. For progesterone-spiked mucus absorbed sponges, 0.5 mM beta-mercaptoethanol (BME) in water or 100% methanol, water alone, or 100% methanol alone was added to replicate sponges for 10 minutes on ice. Sponges were then placed into spin assemblies with pre-weighed 2 mL cryovials, as previously described [37]. Sponges were centrifuged at 20,000 x g at 4°C. Cryovials were then post-weighed and control sponges with elution buffer only were utilized to calculate input or secretion volume, as previously described [37]. Eluted samples were then placed into a cold evaporation chamber and centrifuged for up to 2 hours until all methanol had evaporated. 100 uL of PBS was then added to each sample.

### Progesterone quantification in control samples

Progesterone levels were quantified in unabsorbed control samples in PBS and eluted samples in PBS using a commercially available enzyme-linked immunosorbent assay (ELISA; Abcam, Cambridge, UK), according to the manufacturer’s instructions.

### Progestin hormone quantification in plasma and cervical secretions

Cervical secretions from visit 1, visit 2 (all contraceptive progestin treatments), and visit 3 (Mirena only), were eluted using 100% methanol and secretion volumes quantified utilizing previously published protocols [37]. Progesterone and progestin levels were quantified using liquid chromatography-mass spectrometry/mass-spectrometry (LC-MS/MS) methodologies [38, 39].

In brief, individual stock solutions for medroxyprogesterone and progesterone (P4), beta-estradiol (β-E2), norgestrel, etonogestrel, and deoxycorticosterone acetate (DOCA), as an internal standard were prepared in methanol. All standards were stored at -20°C and stock solutions were stable for at least 3 months. Six calibration standards were prepared at concentrations of 0, 0.05, 0.1, 0.5, 1, 5, and 10 ng/mL. High performance liquid chromatography (HPLC) mobile phases were as follows: Mobile phase A was 0.1% formic acid in HPLC grade water (Fisher). Mobile phase B was 100% Methanol (Optima Grade, Fisher).

Two hundred microliters of either serum or mucus sample was spiked with 10 μL of internal standard, 50 ng/mL (DOCA) and 1mL 100 mM potassium phosphate, pH 9 then extracted with 4 mL of pentane. After centrifugation, the organic phase was collected and the extraction was repeated as above with 2 mL pentane. The organic phases from the first and second extraction were combined and subsequently dried under a stream of nitrogen gas. The dried samples were reconstituted in 100uL HPLC water/methanol (50/50). The samples were then transferred into injection vials for analysis by LC-MS/MS.

Analyses were performed on an ABI/Sciex 4000QTrap coupled with a Shimadzu 20 Series HPLC. Hormones were separated using a Zorbax XDB-C8, 4.6 x 150mm, 5um (Agilent) paired with a matching guard column. The mobile phase gradient was as follows: 60% B for 1 minute, linear to 90% B over 8 minutes, column wash at 90% B for 4 minutes, column re-equilibration at 60% B for 4 minutes resulting in a total run time of 17 minutes. The flow rate of 0.8 mL/min was held constant throughout the method. The column oven was maintained at 32^o^ C. Ions were generated in positive mode using atmospheric chemical ionization. Ions used for quantitation were as follows: MPA (327.1), P4 (297), β-E2 (201.9), norgestrel (108.9), etonogestrel (109.2), and DOCA (331.2).

### Statistical methods

Wilcoxon matched-pairs signed rank tests were performed using Prism software (v7.0; GraphPad, San Diego, CA, USA) to compare cervical and serum levels of each progestin. A p value < 0.05 was considered significant.

## Results

### Optimization of hormone elution from ophthalmic sponges

**Fig 1:**
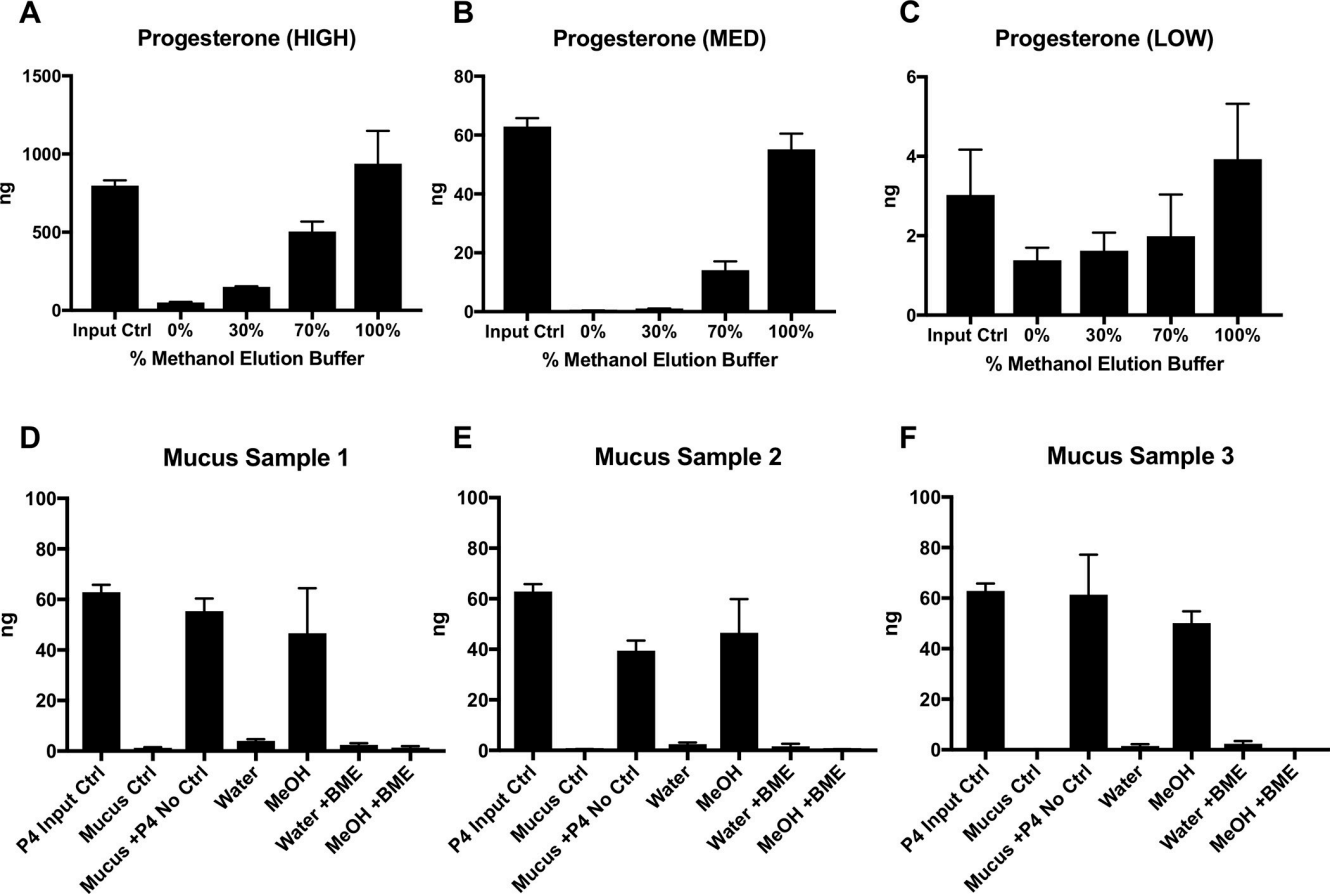
Optimization of progesterone elution from ophthalmic sponges. (A-C) High (A; 500 ng), medium (B; 50 ng), and low (C; 5 ng) levels of progesterone were spiked onto ophthalmic sponges, and eluted with varying percentages of methanol in water. (D-F) 50 ng progesterone was spiked into 25 uL mucus from three subjects and then absorbed onto ophthalmic sponges. Sponges were eluted with water or methanol, with or without 0.5 mM beta mercaptoethanol (BME). Progesterone concentrations were measured by ELISA. Bars represent the mean progesterone levels ± standard deviations (SDs). Data are representative of 3 independent experiments each performed in duplicate. Student t tests were performed to compare input control levels to levels eluted from ophthalmic sponges. No statistically significant differences were noted between any groups.

In order to accurately measure progestin levels in cervical secretions, we first investigated which elution method would optimize hormone retrieval from Merocel ophthalmic sponges. Hormone molecules are large ringed structures that can become lodged within sponge material or bind to mucus in cervical secretions, making accurate quantitation difficult. Since we aimed to quantify progesterone and progestin using LC-MS/MS methodology, and hormonal structures are typically soluble in organic alcohols, we initially investigated methanol as an elution buffer. We first utilized progesterone for these assays, since it is similar in structure and function to the progestins measured in our exposure study, and there is a readily available, sensitive and reproducible commercial ELISA for progesterone quantification. High, medium, and low quantities (500 ng, 50 ng, and 5 ng) of progesterone were spiked onto ophthalmic sponges and then frozen at -80°C to reproduce the methods used for cervical secretions from our patient cohort. Sponges were then thawed on ice and placed into elution chambers, as previously described [37]. Varying dilutions of methanol in water (100%, 70%, 30%, or 0%) were added to each progesterone-spiked sponge, followed by centrifugation, cold evaporation of methanol, and resuspension in PBS. Progesterone input controls, also in PBS, and eluted samples were then assayed for progesterone levels by ELISA. At high, medium, and low progesterone levels (Fig 1A, 1B and 1C, respectively), 100% methanol elution buffer yielded progesterone levels closest to input control levels. These observations suggested that 100% methanol was optimal for eluting progesterone, and likely other ringed hormonal molecules from ophthalmic sponges.

Next, we investigated whether mucus from the female reproductive tract could hinder the elution of steroid hormones from ophthalmic sponges. We used 3 control CVM samples from normally cycling women unexposed to exogenous progestins and added a known quantity (50 ng) of progesterone to each sample. Half of these samples were then absorbed onto ophthalmic sponges and stored at -80°C. Since mucus may not have efficiently eluted in methanol due to the complex structures of the mucin proteins [36, 40, 41], we investigated whether BME might be useful to break the disulfide bonds in the mucin proteins and optimize progesterone elution from sponges. Water, water with BME, 100% methanol, or 100% methanol with BME was added to progesterone-spiked mucus absorbed sponges. Sponges were then centrifuged, exposed to cold evaporation and resuspended in PBS. Eluted samples, input controls, and unabsorbed mucus controls were assayed for progesterone using ELISA. Similar to results observed in Fig 1A–1C, 100% methanol without BME appeared to optimize progesterone retrieval from ophthalmic sponges in all mucus samples (Fig 1D, 1E and 1F). Interestingly, addition of BME to methanol abrogated the efficient elution of progesterone from the sponges. Taken together, 100% methanol appeared to be the optimal solvent for eluting progesterone from mucus samples absorbed onto collection sponges.

**Fig 2:**
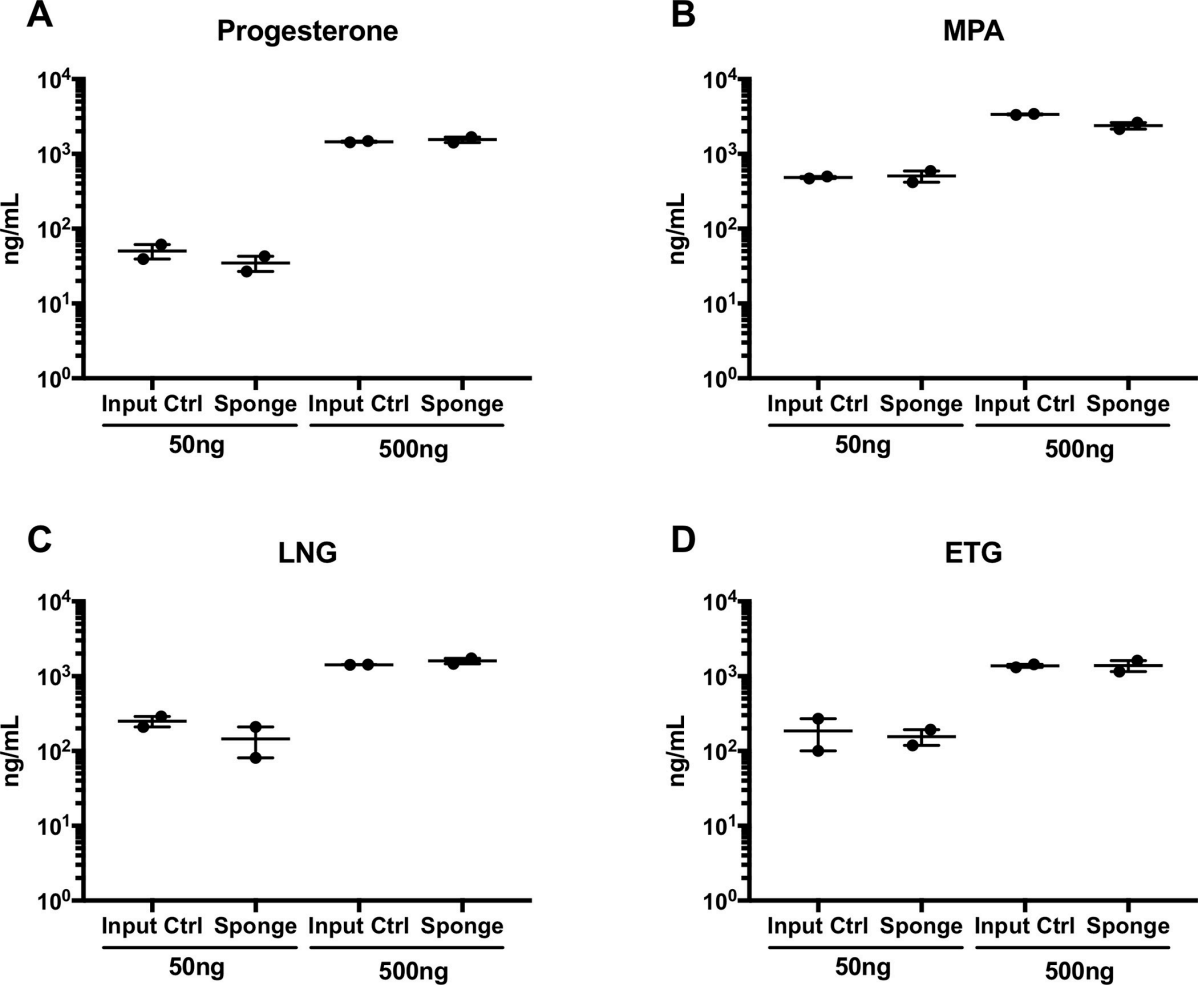
Progestins in cervical mucus can be efficiently retrieved from ophthalmic sponges. Fifty and 500 ng of progesterone and progestins were spiked onto ophthalmic sponges and eluted with 100% methanol. Progesterone (A) and progestin (B-D) values were measured via LC-MS/MS methodologies. Data are representative of 2 independent experiments. Student t tests were performed to compare input control levels to levels eluted from ophthalmic sponges. No statistically significant differences were noted between any groups.

Finally, we utilized these optimized methods to verify that those progestins to be quantified from our patient cohort samples, MPA, LNG, and ETG, were all efficiently eluted and quantifiable using the same methodologies. We therefore spiked two known concentrations (50 ng, 500 ng) of progesterone and the three progestins to be measured onto ophthalmic sponges. Hormones were eluted from sponges using 100% methanol, as determined in Fig 1. Progesterone and progestin levels were then quantified in eluted and unabsorbed control samples in methanol using LC-MS/MS (Fig 2). No statistical differences were seen between measured concentrations of unabsorbed stock controls and levels measured in eluted sponge samples for any of the hormones measured (Fig 2A–2D). 100% methanol was therefore used to elute and quantify progestins from cervical sponges collected from our patient cohort.

### Progestin concentrations in plasma and cervical secretions

**Fig 3:**
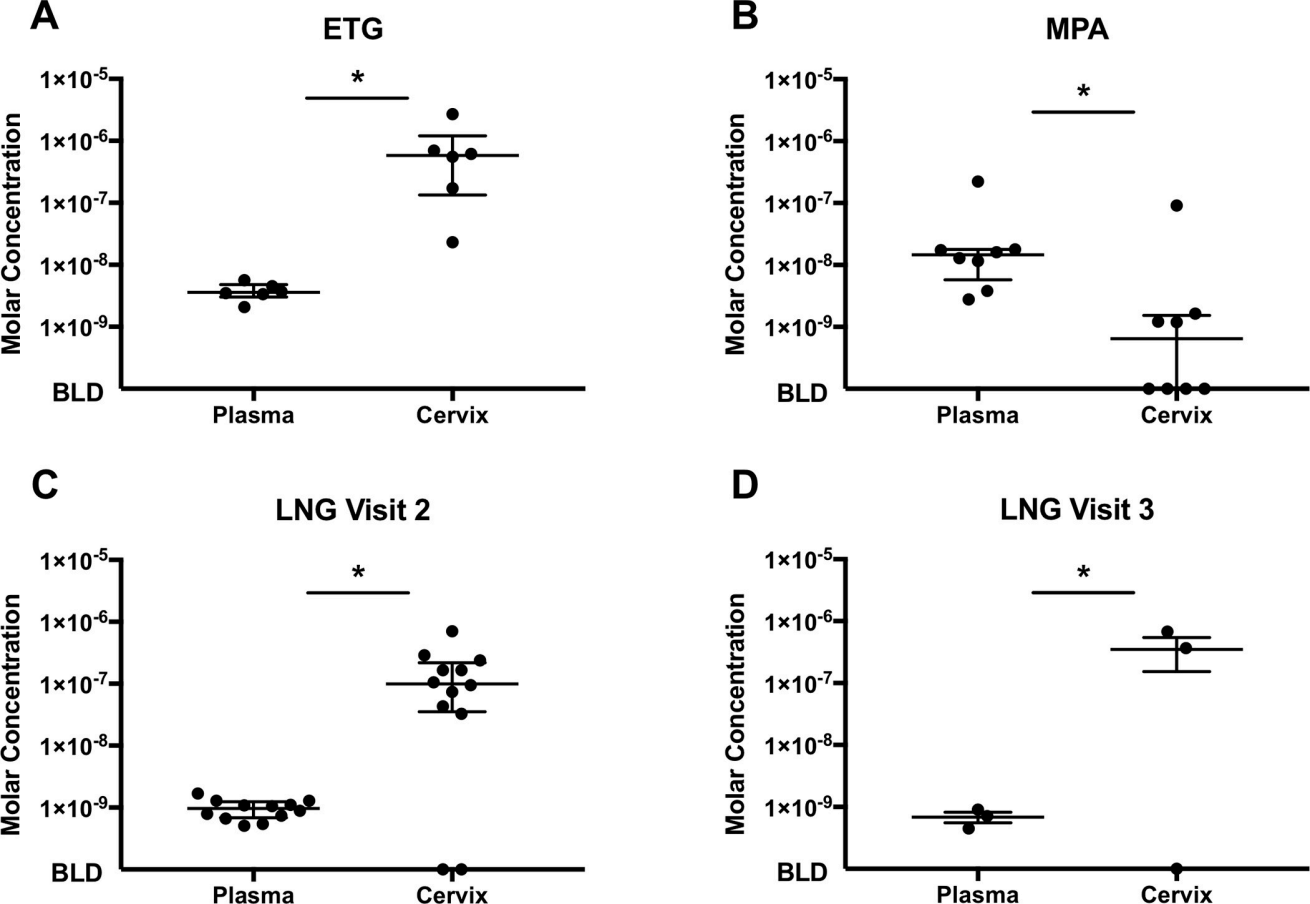
Locally delivered progestins yield higher concentrations in the cervix compared to systemically delivered MPA. Visit 1 (pre-progestin treatment), Visit 2 (post-progestin treatment), and Visit 3 (6 month post-LNG only) samples were assayed for their respective progestins in cervical and plasma specimens using LC-MS/MS methodology. Cervical specimens were eluted from ophthalmic sponges using 100% methanol and concentrations of progestins measured in the secretion volumes. Wilcoxon matched-pairs signed rank tests were performed to compare plasma and cervical levels of each progestin for each group with * denoting p<0.05. (A) ETG (n = 6, p = 0.03), (B) MPA (n = 8, p = 0.008), (C) Visit 2 LNG (n = 12, p = 0.002), (D) Visit 3 LNG (n = 3, p = 0.50). There were no detectable progestins in Visit 1 samples (data not shown).

The levels of progestins delivered locally, ETG via the vaginal ring and LNG via an IUS, were higher in cervical secretions when compared to levels measured in plasma samples from the same women (Fig 3A, 3C and 3D). Median levels of ETG were 5.8x10^-7^M (IQR = 1.1x10^-6^) in cervical secretions with a range of 2.3x10^-8^M to 2.7x10^-6^M. Median plasma ETG levels were 3.6x10^-9^M (IQR = 1.8x10^-9^) with a range of 2.1x10^-9^M to 5.7x10^-9^M (p = 0.03). Median levels of LNG were 1.0x10^-7^M (IQR = 1.8x10^-7^) in cervical fluid from visit 2 samples (1 month post treatment) (range ND (n = 2) to 7.0x10^-7^M) and 3.7x10^-7^M (IQR = 6.8x10^-7^) from visit 3 samples (approximately 6 months post treatment) (range of ND (n = 1) to 6.8x10^-7^M). Median plasma levels of LNG were 9.7x10^-10^M (IQR = 5.6x10^-10^) from visit 2 samples (range of 5.1x10^-10^M to 1.7x10^-9^M) and 7.1x10^-10^M (IQR = 4.6x10^-10^) in visit 3 samples (range of 4.5x10^-10^M to 9.0x10^-10^M) (p = .002, p = 0.5, respectively). In contrast, levels of MPA, delivered as a systemic intramuscular injection, were higher in the plasma (median levels 1.5x10^-8^M (IQR = 1.2x10^-8^), range of 2.8x10^-9^M to 2.2x10^-7^M) when compared to levels measured in the local secretions (median levels 6.4x10^-10^(IQR = 1.4x10^-9^), range of ND (n = 4) to 9.1x10^-8^M) (p = 0.008) (Fig 3B).

## Discussion

The WHO and the joint United Nations Program on HIV/AIDS (UNAIDS) recently made the elimination of maternal-to-child transmission of HIV one of their primary goals [42]. Achieving this objective will rely on efforts to provide safe and effective contraceptive options to women at high risk for HIV acquisition. LARCs that are discrete, woman-initiated and characterized by low user compliance failure rates are particularly valuable. DMPA is a progestin-only option that has provided great contraceptive efficacy for women, making it the 4^th^ most common LARC used worldwide [43]. Great concern has been raised over use of DMPA in the last few decades, however, due to consistent results in non-human primates demonstrating that use of this particular LARC increases the risk of simian immunodeficiency virus (SIV) transmission in the FGT [44, 45], increases post-infection viremia levels [46], and increases cervical shedding of HIV infected cells [47, 48]. Furthermore, epidemiological studies have also noted increased HIV transmission rates among high risk women who use DMPA [5, 49–51] [8]. Cessation of DMPA use in high risk women without substituting alternative effective contraceptive options could increase unwanted pregnancy rates as well as mother-to-infant HIV transmission should a woman become infected [52–54]. The public health community cannot afford to continue to promote contraceptive options, such as nonoxynol-9 [55] and DMPA, that place undue risk on vulnerable populations. As such, the WHO has recently altered their recommendations on use of DMPA in women at high risk for HIV acquisition from category 1 in which there are no restrictions on use to category 2 in which the advantages outweigh the risks and the contraceptive can generally be used, however women considering DMPA use must first be provided information on the associated risks with HIV acquisition [9]. Therefore, safe and effective LARC alternatives must be identified and made available to women at risk for contracting HIV.

*In vitro* and *in vivo* comparisons of the different progestin-containing contraceptive options and their influence on sexual transmission of HIV are plagued by many issues. These include the variety of progestin subtypes included in female contraceptives and their respective hormone receptor binding profiles, differences in the dosages and routes of progestin administration, and the use of formulations that are combined with contraceptive estrogens. Particularly striking among these factors is the remarkable paucity of information on local FGT levels of exogenous hormones that could allow accurate *in vitro* modeling. To date, numerous *in vitro* studies have been performed using serum levels of progestins as clinical surrogates. Given that some progestins are delivered systemically (e.g., DMPA) while others are delivered into the lumen of the FGT (e.g., LNG via an IUS and ETG via vaginal ring), it is likely that serum levels do not reflect the local progestin concentrations to which epithelial cells and underlying tissues are exposed for some or all methods. This is a particularly important factor to consider for *in vitro* modeling because the contraceptive and non-contraceptive effects of each progestin are concentration dependent [23, 24], and using an inappropriate concentration of progestin in culture could yield results that do not reflect progestin-mediated effects in the FGT *in vivo*. Thus, the purpose of this study was to determine the levels of several commonly used contraceptive progestins in cervical secretions and compare these to plasma levels in order to establish proper *in vitro* modeling conditions for future investigations.

We focused on the cervix in this study because non-human primate studies indicate the endocervix as a major site of SIV transmission [56]. This is postulated to be the result of a vulnerable single-cell thick epithelium combined with the relatively high number of underlying target immune cells at this site [57], a combination that is unique in the human FGT. We first optimized methodologies to effectively elute and measure steroid hormones from ophthalmic sponges. We determined that 100% methanol was the optimal elution solvent for efficient retrieval of progesterone and contraceptive progestins from mucus absorbed onto ophthalmic sponges.

We then used this methodology on ophthalmic sponge and plasma samples collected pre- and post- exposure to several progestin-containing LARCs *in vivo*. We observed that levels of locally delivered progestins, LNG delivered via an IUS and ETG via a vaginal ring, were approximately 100–200 times higher in the local cervical secretions when compared to the periphery. Levels of MPA after delivery by intramuscular injection, however, were >10 fold higher in the plasma compared to cervical secretions. Interestingly, approximately 50% of samples collected from women treated with DMPA had unmeasurable levels of MPA in their cervical secretions. This is not unexpected, since MPA is delivered systemically, so epithelial cells and other tissues are exposed to this hormone basolaterally via the bloodstream rather than apically within the FGT as with contraceptive progestins delivered via a locally-placed device. In the latter scenario, the measurement of locally-delivered progestin concentrations in samples obtained from the FGT lumen, where delivery occurs, is feasible, reproducible and more physiologically relevant to HIV transmission across these epithelial barriers than peripheral measurements. While significantly more variability is encountered when measuring systemically-delivered progestin levels in luminal samples, these measurements also provide improved physiologic application and enable more informed modeling of *in vitro* investigations. In short, it is important to utilize progestin concentrations in a polarized manner when modeling progestin effects on epithelial cells or tissue explants *in vitro*. Specifically, the working concentrations utilized *in vitro* should reflect median hormone levels for both peripheral and local concentrations for MPA, 10^-8^M MPA at the basolateral epithelial surface and 10^-10^M MPA at the apical surface of polarized *in vitro* models. For investigating the effects of locally delivered progestins on epithelial cells or tissue explants, the basolateral surface should be exposed to median peripheral concentrations, *e*.*g*. 10^−9^ M ETG, 10^-10^M LNG, and the apical epithelial surface exposed to median luminal concentrations at, *e*.*g*. 10^-7^M ETG, 10^-8^M LNG. Similarly, when performing experiments investigating the influence of progestins on peripheral blood cells, peripheral progestin concentrations, *e*.*g*. 10^-8^M MPA, 10^−9^ M ETG, 10^-10^M LNG, should be replicated, as these represent the approximate median hormone levels to which circulating leukocytes are exposed. These findings provide context for previously published *in vitro* progestin studies and their observations. For instance, by using a range of exposure levels, Sampah *et al*. demonstrated an increase in HIV infection of MPA exposed target cells using concentrations representative of those found in blood (approximately 10^-9^M) as well as the cervical environment (approximately 10^-10^M). This suggests that MPA may influence infection and replication of virus in the local tissues as well as the periphery [58].

Overall, the findings in this study provide a valuable guide for *in vitro* and *in vivo* progestin exposure study designs. Our optimized methodologies can be utilized by others to elute steroid hormones from ophthalmic sponges for further investigation of the local genital tract milieu in women who have initiated progestin treatments. Furthermore, the progestin levels reported here for local secretions and plasma inform the community on study design for epithelial, tissue explant, or peripheral blood cultures in the context of synthetic progestins that depend upon both the type of progestin and delivery method. Such study design parameters are essential, as the immunological and contraceptive effects of progestins are concentration dependent and uninformed design could yield results that are not applicable to *in vivo* outcomes. Our results should help to uncover specific, concentration-dependent, progestin-mediated mechanisms by which systemic and local exposure to DMPA increases HIV infection and/or replication in target cells. Moreover, these results will facilitate appropriate *in vitro* testing of alternative contraceptive progestin replacement options and thereby guide physicians in making safe contraception recommendations for women at high risk for HIV acquisition.

## Supporting information

S1 File BucknerManuscriptRawData. (XLSX)
